# Case Report: Pericardial Tamponade and Hemothorax After Superior Vena Cava Filter Removal

**DOI:** 10.3389/fcvm.2022.863732

**Published:** 2022-06-03

**Authors:** Xueping Zeng, Jin Zhou, Qidi Zhou, Zhigang Huang

**Affiliations:** ^1^Department of Emergency, Peking University Shenzhen Hospital, Shenzhen, China; ^2^Department of General Practice, Peking University Shenzhen Hospital, Shenzhen, China

**Keywords:** superior vena cava filter (SVCF), upper extremity deep venous thrombosis (UEDVT), pericardial tamponade, hemothorax, pulmonary embolism (PE)

## Abstract

A 67-year-old male patient was admitted to receive surgical treatment because of lumbar spinal stenosis. Ultrasonography showed a thrombus in the middle and lower segment of the left internal jugular vein. Superior vena cava filter implantation was performed and removed on day 7 after its implantation. The patient suddenly had dyspnea, and his blood pressure decreased 9 h after filter removal. Examinations showed pericardial tamponade and hemothorax. In addition, aortic dissection occurred approximately 20 days after superior vena cava filter removal. This case aimed to provide information to clinicians about the complications of the implantation and removal of superior vena cava filter implantation. The safety and possible complications of superior vena cava filter implantation and removal should be evaluated to identify their actual clinical benefit and cost-effectiveness ratio.

## Background

In recent years, with the development of clinical intervention technology, the vena cava filter (VCF) has been widely used in patients with deep venous thrombosis (DVT). Acute pulmonary embolism (PE) is the main cause of sudden death in patients with DVT. Previous studies have shown that the PE incidence in patients with DVT of the lower extremities is approximately 45% (1), and the risk of PE due to upper extremity DVT (UEDVT) can reach up to 5%−10% (2).

At present, inferior vena cava (IVC) filters have satisfactory clinical outcomes in preventing the development of PE and in reducing its associated mortality rates (3). In contrast, the placement of a VCF in the superior vena cava (SVC) for preventing PE caused by UEDVT is controversial (4). However, using SVC filters (SVCFs) as a safe, feasible, and effective alternative method can prevent PE in patients with anticoagulant contraindications, thrombolytic failure, and preoperative UEDVT (5). Filter placement in SVC is technically more challenging than IVC due to the relatively small area for filter insertion and displacement (6). The complications of SVCF implantation are classified as early and late. The early complications include incorrect and tilted filter implantation position, poor patency and displacement, and puncture point complications. Meanwhile, the late complications include PE and DVT, vena cava obstruction, filter fatigue fracture, and perforation. Other rare complications include pneumothorax, hemothorax, arteriovenous fistula, air embolism, and pericardial tamponade (7, 8).

According to statistics, in recent years, the annual incidence of IVC filter-related complications is approximately 1.5% (9). Reports about the use of SVCF in treating patients with UEDVT are limited, and studies into the safety and efficacy of SVCF are even scarcer. Hence, the use of SVCF in clinical practice remains controversial. The current study assessed SVCF-related complications by analyzing the data of a patient who presented with cardiac tamponade and hemothorax after SVCF removal.

## Case Presentation

A 67-year-old male patient with complaints, such as repeated lumbago and radiating pain in both lower limbs for 2 years and dizziness for more than 1 month, was admitted to our hospital. Lumbar magnetic resonance imaging (MRI) showed L4/L5 intervertebral disc herniation with spinal canal stenosis. The patient was admitted to the department of spinal surgery for lumbar spinal stenosis and received elective surgical treatment.

**Figure 1:**
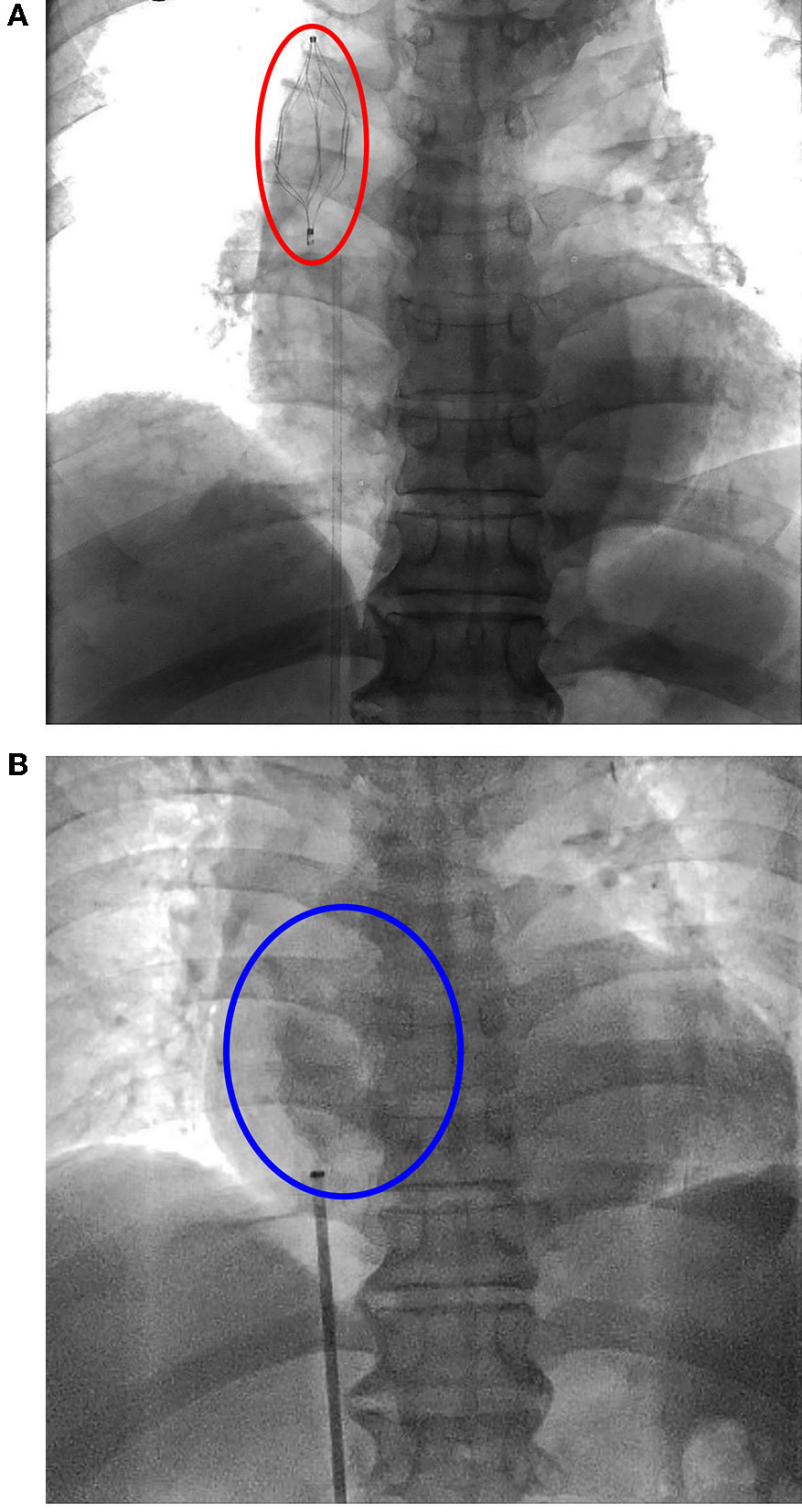
**(A)** Superior vena cava filter (SVCF) implantation. Vena cava filter (red oval) was on the position. **(B)** Superior vena cava filter removal. There was no spillover of contrast agent (blue oval).

**Figure 2:**
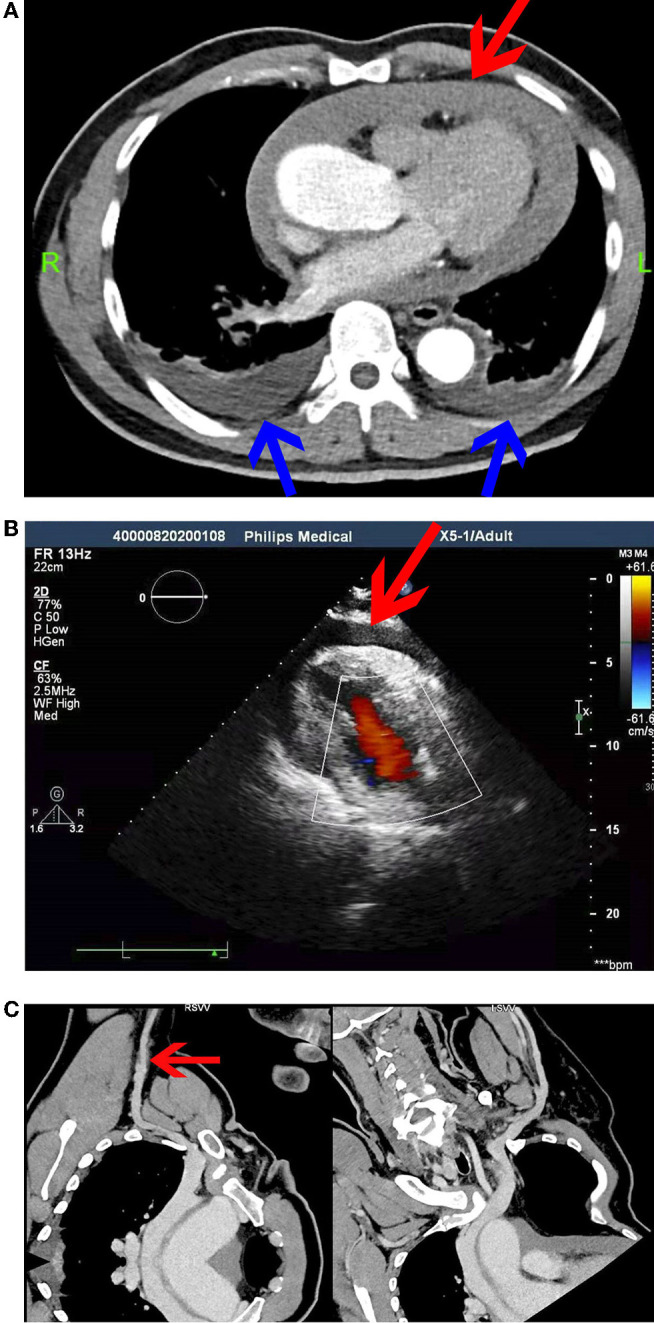
**(A)** Computed tomographic pulmonary angiography (CTPA). Pericardial effusion or hemorrhage (red arrow) and a small volume of pleural effusion (blue arrow) were observed. **(B)** Echocardiography at 12 h after SVCF removal. **(C)** Computed tomographic venography (CTV) of the upper limb vein. The right axillary vein was not filling well (red arrow).

Echocardiography after admission revealed no evident abnormalities. Bilateral cervical vascular color ultrasonography showed a thrombus in the middle and lower segment of the left internal jugular vein. Considering that venous thrombus might fall off, causing severe thromboembolism events, such as PE and post-embolization syndrome, SVCF implantation was performed under digital subtraction angiography on day 3 after admission (Figure 1A). The right femoral vein approach was adopted, and the implantation process was smooth. The filter was placed at the upper end of the SVC in good position and shape, and the blood flow in the filter was patent. The patient was safely returned to the ward after surgery and received anticoagulation therapy with low-molecular-weight heparin. The patient underwent spinal surgery after SVCF implantation. SVCF removal was performed on day 7. Intraoperative re-examination showed that the upper vena cava was smooth (Figure 1B). The filter was entirely removed without damage, and the patient was returned safely to the ward. Nine hours after SVCF removal, the patient got off the bed and suddenly had dyspnea and fatigue. The electrocardiographic monitor showed that the patient's heart rate rose to 100–110 beats per minute, and his lowest blood pressure was 56/49 mmHg. His level of consciousness was normal, and the rest of the physical examination showed no abnormalities. Emergent computed tomographic pulmonary angiography (CTPA) revealed that the ascending aorta widened with a diameter of approximately 5 cm. There were no signs of pulmonary embolism. However, pericardial effusion or hemorrhage and a small volume of pleural effusion were observed (Figure 2A). In addition, an echocardiogram showed a medium liquid dark area in the pericardial cavity, 17 mm in front of the anterior wall of the right ventricle, 16 mm behind the posterior wall of the left ventricle, 9 mm at the apex, 17 mm outside the lateral wall of the left ventricle, and 14 mm outside the lateral wall of the right ventricle (Figure 2B). Emergency blood routine tests and dynamic monitoring of vital signs showed no evident signs of active bleeding. Based on these findings and clinical manifestations, the patient was diagnosed with pericardial tamponade and bilateral pleural effusion after hemopericardium. After providing oxygen therapy and rapid fluid infusion, the patient's heart rhythm was 83 beats per minute and blood pressure was up to 110/80 mmHg, and his symptoms were relieved.

**Figure 3:**
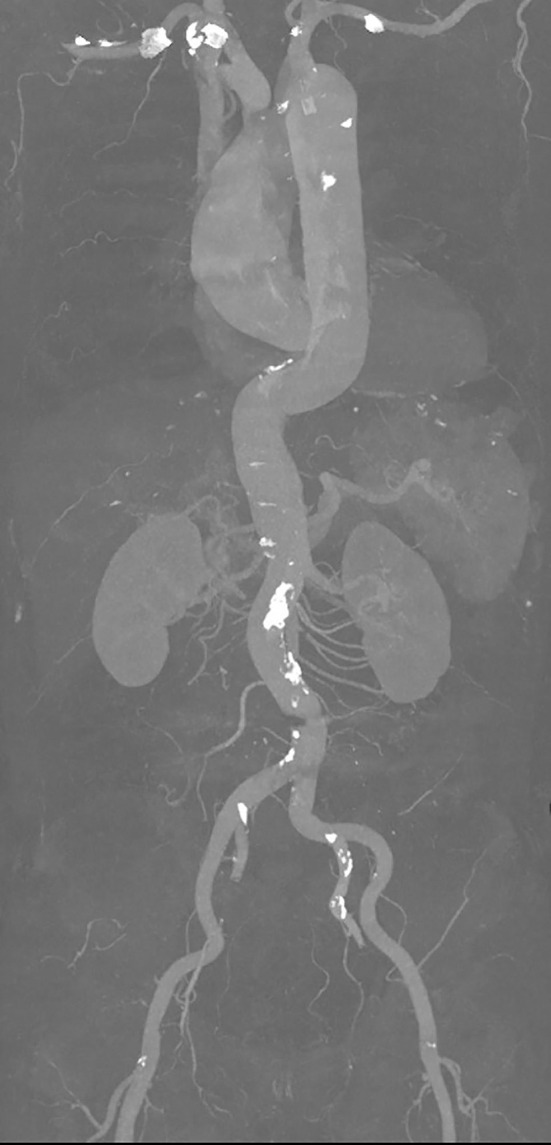
Aortic dissection.

The patient was transferred to the intensive care unit (ICU) for further treatment. During his admission, a dynamic re-examination of the bedside echocardiogram showed that pericardial effusion was not absorbed significantly, and the patient still had chest tightness and discomfort in the precordial area. On day 4 after SVCF removal, computed tomographic venography (CTV) of the upper limb vein was performed, and the results showed that the right axillary vein was not filling well. Thus, thrombosis was highly suspected (Figure 2C). Re-examination of the bedside echocardiography showed a liquid dark area in the pericardium, 13 mm in front of the anterior wall of the right ventricle, 16 mm behind the posterior wall of the left ventricle, 18 mm at the apex, 22 mm outside the lateral wall of the left ventricle, and 20 mm outside the lateral wall of the right ventricle. The volume of pericardial effusion increased after 3 days. Further, his blood pressure decreased to 93/58 mmHg, and his heart rate was 100 beats per minute. Chest tightness and dyspnea were more evident. Pericardiocentesis and catheter drainage were performed on the same day. After 600 mL of non-coagulated blood was drained, bedside echocardiography revealed a lower volume of pericardial effusion, which was observed 10 mm behind the posterior wall of the left ventricle, 7 mm at the apex, and 16 mm outside the lateral wall of the left ventricle. Chest tightness and dyspnea were significantly relieved. Further, his blood pressure increased to 138/100 mmHg, and his heart rate was 78 beats per minute. Simultaneously, thoracic ultrasonography revealed that bilateral pleural effusion progressed, and bilateral thoracentesis and catheter drainage was performed on day 5 after SVCF removal. Echocardiography after catheterization showed that the maximum anteroposterior diameter of the dark area at the anterior wall of the right ventricle during the diastolic period was approximately 5 mm, and the systolic function of the left ventricle was normal. Further, there were traces of pericardial effusion. Thoracic ultrasonography showed no evident effusion in the bilateral pleural cavity. The drainage tube was removed 4 days after pericardium drainage and bilateral thoracic drainage, and 2,900 mL of the pericardium and bilateral pleural effusion was drained. The patient was discharged after his condition stabilized.6 days after discharge, the patient had sudden difficulty breathing without evident chest pain. Computed tomography angiography (CTA) of the whole aorta revealed aortic dissection (Stanford type A), which involved the aortic valve (Figure 3). Emergency surgery revealed a rupture of the initial part of the ascending aorta on the right posterior area. The patient's postoperative recovery was satisfactory, and the prognosis was good.

## Discussion

The annual incidence of venous thromboembolism is high (10), and anticoagulation is the standard treatment for patients with this condition. VCF is an alternative treatment for patients with contraindications to anticoagulation or as adjuvant therapy for patients with embolism after anticoagulation. With the widespread application of technology, different complications occur after VCF implantation. These complications are occasionally fatal (11). The development of complications after SVCF placement is rare. However, they may be more severe than IVCF. In strut perforation, there is no barrier in the mediastinum similar to the retroperitoneal cavity to protect surrounding organs. Although strut perforation in IVC does not commonly cause any complications, SVC wall perforation may damage the aorta, pulmonary artery, lung, heart, or pericardium (5, 12, 13). Nicholson et al. (14) reported three cases of severe arrhythmia and pericardial tamponade caused by rod fracture. One patient died, and the other two underwent emergency heart surgery, during which the broken filter fragments were removed.

A study showed that the degree of vena cava injury is correlated with the time of VCF implantation (15). When the filter insertion time is longer, the inner membrane grows more tightly, and the filter cannot be recovered. In this report, the Aegisy VCF (LifeTech Scientific Co., Ltd., Shenzhen, China), a nickel-titanium alloy filter, was used, and the implantation time was 7 days. The patient suddenly had dyspnea, and his blood pressure decreased 9 h after filter removal. An implanted filter may be fixed and can adhere to the SVC wall. The pericardial reflex position where the SVC enters the right atrium was damaged due to excessive force during filter removal. Then, the blood enters the pericardium from the laceration. Moreover, anticoagulant therapy with heparin was used for filter implantation and postoperative treatment. Then, the blood was challenging to coagulate, and bleeding was not easy to control, which led to the development of pericardial effusion and tamponade. Simultaneously, the mediastinal pleura was damaged after SVCF removal, thereby resulting in bilateral hemothorax. In addition, because the pericardium expands and compresses local tissues, the venous pressure in the pleural capillaries increases, and lymphatic reflux is blocked, thereby causing the production of bilateral chest leakage. This phenomenon further aggravates the clinical symptoms of chest tightness and dyspnea. Although the patient underwent computed tomographic venography (CTV) of the upper limb veins, there was no evident venous filling defect. However, the possibility of vascular wall injury during filter removal could not be ruled out. In addition, type A aortic dissection occurred about 20 days after SCVF removal. The rupture was located in the initial part of the ascending aorta on the right posterior area. Hence, aortic membrane injury during filter removal was not ruled out. Meanwhile, as time had passed and there were fluctuations in blood pressure, dissection was performed.

## Conclusion

Herein, the occurrence of complications after superior vena cava filter removal was reported, which was correlated with pericardial effusion, mediastinal pleural, and aortic membrane injury caused by filter implantation. The current study aimed to provide information to clinicians about the complications of not only SVCF implantation but also SVCF removal. Based on the current data, SVCF implantation is a safe and effective alternative method for preventing PE caused by DVT of the upper limbs if anticoagulant therapy is not feasible. However, the safety and possible complications of SVCF implantation and removal should be evaluated to identify the actual clinical benefit and cost-effectiveness ratio. Nevertheless, more clinical data are required to assess the long-term morbidity and mortality of SVCFs and to confirm the role and clinical value of VCFs.

## Data Availability Statement

The original contributions presented in the study are included in the article/supplementary material, further inquiries can be directed to the corresponding author.

## Ethics Statement

All clinical data in this case report were collected with the consent of the patient. Written informed consent was obtained from the patient for the publication of this report and for the publication of any potentially identifiable images or data included in this article.

## Author Contributions

ZH provided intraoperative care and designed the manuscript. QZ was the physician of record. JZ collected the information of patients. XZ organized the information of patients and edited the manuscript. All authors contributed to manuscript revision, read, and approved the submitted version.

## Funding

This study received a grant from the Key Laboratory of Emergency and Trauma, Ministry of Education (KLET-202001).

## Conflict of Interest

The authors declare that the research was conducted in the absence of any commercial or financial relationships that could be construed as a potential conflict of interest.

## Publisher's Note

All claims expressed in this article are solely those of the authors and do not necessarily represent those of their affiliated organizations, or those of the publisher, the editors and the reviewers. Any product that may be evaluated in this article, or claim that may be made by its manufacturer, is not guaranteed or endorsed by the publisher.
